# The Moral Treatment, Hygiene, and Education of Idiots, and of Other Children Backward or Retarded in Their Development, Agitated with Involuntary Movements, Weak, Dumb, but Not Deaf, Stammering, &c.

**Published:** 1847-07

**Authors:** 


BRITISH AND FOREIGN

### MEDICAL REVIEW,

FOR JULY, 1847.

### PART FIRST.

### Analytical and Critical Reviews.

### Art. I.

1. *Traitement Moral, Hygiène et Education des Idiots et des autres Enfants arriérés ou retardés dans leur développement, agités de Mouvements involontaires, débiles, muets non-sourds, bègues, &c.* Par Edouard Séguin.—*Paris,* 1846.

*The Moral Treatment, Hygiene, and Education of Idiots, and of other Children backward or retarded in their development, agitated with involuntary Movements, Weak, Dumb, but not Deaf, Stammering, &c.* By Edward Séguin.—*Paris,* 1846. 8vo, pp. 734.

2. *Remarks, Theoretical and Practical, on the Education of Idiots and Children of Weak Intellect.* By W. R. Scott, ph.d., Principal of the West of England Institution for the Education of the Deaf and Dumb. —*London and Exeter,* 1846. 8vo, pp. 46.

M. Séguin informs us in his introduction that he has studied idiotcy for ten years, that owing to a report by Pariset on his method, and to the encouragement and approbation of the Academy of Sciences, he was intrusted with the education of the young idiots in the Paris hospitals;—that his work is entirely new, for though encouraged by Itard and Esquirol, he was compelled to find his resources in himself;—that in his investigation he was forced to inquire into the plans of education, both intellectual and physical, most in vogue, and to weigh their merits by applying them to practice. We learn from the book itself, that M. Séguin is not a physician, but a teacher;—that, instigated by M. Itard, he has devoted himself wholly to the education of idiots; and that to effect this, the teacher must carry out his plans himself with the most laborious painstaking. The education of beings so imperfect in body as well as in mind, taught him in language not to be mistaken that the due performance of the healthy bodily functions is essential to the action of the mind, and that every complete system of education embraces physical as well as intellectual and moral training. The rules of Hygiene must therefore be included in any complete treatise on Education. The relative importance of M. Séguin's views are in accordance with the attention he has bestowed on the various branches of the subject. The descriptions of the habits, and outward peculiarities of idiots, as well as their mental characteristics, are original and masterly; so are the rules for physical and moral training; the medical portion, and especially that which might be enlightened by pathological anatomy is meagre and unsatisfactory, whilst the hygiene and physiology are but common-place. Non omnia possumus omnes. To be a practical physician requires the exclusive devotion of a life, and we do not expect, from one who lays himself out for the work of education, anything more than a general acquaintance with subjects beyond his own province.

Dr. Scott's pamphlet, although creditable both to the head and heart of the author, contains nothing of importance not found in greater detail in M. Séguin's book. We shall not, therefore, take any special notice of it in the following pages. We had prepared an analysis of the description of idiotcy, its varieties, its physiological and psychological symptoms, its causes, diagnosis, and prognosis, and the rules for hygiene; but on finishing the whole, we found that the article was so bulky as to be beyond the space we could devote to the subject. The only mode of curtailing it seemed to be to omit this preliminary portion, and to insert only the analysis of the plan of education, physical, moral, and intellectual, for this plan is so dependent in its parts that it is impossible to omit any link without sacrificing its practical use. We regret omitting his descriptions of idiotcy, for they come from an observant and practised eye, guided by a thoughtful mind. Every page shows that the writer has lived with his subject, and has given to it all the devoted attention of an energetic nature. He writes from his own stock, not loose generalities, the result of reading. His style is not classical, but on the other hand it has a freshness better than mere tame correctness.

Before entering on the subject of education, there are, however, two or three points to be alluded to. The first is the definition of idiotcy.

*What then is idiotcy?* Idiotcy is a disordered state of the nervous system, owing to which there is no regular command of the will over all or part of the organs and faculties of the child, who is guided by his instincts, and cut off from the moral world. The type of an idiot (an ideal idiot?) is an individual who knows nothing, wishes nothing, and can do nothing, and every idiot approaches more or less to this *summum* of incapacity.

*Psychological symptoms of idiotcy.* Though confined in narrow limits the idiot still has sensations, sentiments, and limited perceptions, manifested by his wants, appetites, tastes, inclinations, desires, repugnances, fears, terrors, preferences, wishes, all expressed in his own way. He gives evidence that he remembers perceptions of his senses, that he can compare these past sensations with present ones, and that he can look forward and judge of the future from the past though this power is very circumscribed. That which is wanting in the idiot is not then distinct perception, as he can distinguish his food; nor internal nor external sensations, nor attention, as he can fix it and his wishes on those things which please him; nor comparison nor judgment, as he can choose among many things that which pleases him most; nor understanding, when he submits to the moral influence of mild or severe language; nor to foresight of his wants and appetites, for though few, yet he will use great patience and ingenuity to gratify them; nor tastes, for although limited to the desire of tearing rags or licking a piece of crockery, they occupy the mind as completely as the taste for dancing or gaming occupies higher minds; nor personal affections or antipathies; nor even will itself in the narrow limits of willing to satisfy his instincts or to gratify his indolence. What then does he want intellectually to resemble the rest of the world?" Il ne lui manque," says M. Séguin, "aucune faculté intellectuelle; mais il n' a pas la liberté nécessaire pour appliquer ses facultés dites intellectuelles à l'ordre des phénomènes moraux et abstraits; il lui manque la synergie, la spontanéité d'où jaillit la volonté morale." By which we take M. Séguin to mean that the idiot has not that freedom or power of the will by which he can of himself originate intellectual or moral actions. The reader will perhaps more clearly comprehend the meaning when he has read the whole article.

Idiotcy is greatly aggravated by neglect. All those cases, without which, children well gifted could not have attained the dignity of manhood, are withheld: no kind forethought, no enlightened affection, no plan of education, no methodical treatment. Poor or rich, with affectionate or careless mothers, they are equally doomed to ignorance and inaction; with no assistance, no amusements, no excitements to thought, feeling, or action? Until left to their instincts, their inertness, their nervous disorders, and filthy and repulsive habits, they become incurable, and growing old prematurely die at thirty. Neglect makes their youth a season of decrepitude which leads them rapidly and painfully to their graves. We must refer to M. Séguin's own pages for his indignant remonstrances against those who thus condemn the ignorant to ignorance, the inert to inertia, the idiot to perpetual idiotcy.

The common opinion that idiotcy is incurable, is false, says M. Séguin. This opinion has arisen, 1st, from the incapacity of medical men to form a judgment on this subject from not having specially studied it, and from their hasty visits, a rapidity which prevents any attentive study, or any attempt at moral treatment by personal means. 2. The unwillingness of parents to vex the idiot by a change of habits which at first make him sad, vexed, or produce tears; for the idiot, though he cannot tell black from white, has always a marvellous intuition as to the degree of ascendancy he exercises over each person. 3. Others become after a time indifferent. 4. Religious prejudices, by which some people regard the idiot as incapable of sin.

But, notwithstanding these impediments, idiots have been made good, active, intelligent, and to a certain point useful to their relatives and to society; and it is impossible to say *à priori* what cases are incurable. The trial must first be made. Neither the smallness of the head, nor the hydrocephalic enlargement are signs on which any opinion as to the result can be formed. The most unpromising cases are those attended with general paralysis, or with hemiplegia, chorea, and epilepsy. But it is only after the failure of assiduous means that even these cases can be pronounced incurable; for proper treatment often ameliorates or removes these complications, and thus benefits the disease.

THIRD PART.—THE EDUCATION OF IDIOTS.

In the education of the blind, and of the deaf and dumb, one sense is substituted for another: the touch instead of the sight in the blind, and the sight instead of the ear in the deaf and dumb; but idiots have senses although dormant. They hear, and do not understand; see, and do not perceive. They are thus disconnected from the outer world, and the principle involved in their education is to awaken their attention. Their senses must be roused so as to convey impressions to their brain by a system of sensorial gymnastics, and their brain must be stimulated so as to receive and to react on these impressions by intellectual gymnastics, both being combined with hygienic regimen. The result is that an idiot inferior in capacity and intelligence to domestic animals, becomes if not restored to society, at least restored to his family, his bad habits are corrected, he is more obedient, more active, in better health, and affectionate to those who have given him their affection and support. whilst others have been enabled to read, write, speak, and occupy themselves readily in many manual occupations. If rude is the result (asks M. Séguin) of a system followed up for ten years only and by one man, with limited means, may not much be hoped for when it is perfected by the experience of many?

Education consists not in improving the memory alone, but in developing all the functions of the body and the faculties of the mind. The education of the idiot should comprehend,—1. The active powers. 2. The intellect. 3. The will, that is the voluntary principle under the guidance of the enlightened conscience. (La spontanéité moralisée.) This is in order of their development. The infant moves and feels before he knows, and he knows before he has the consciousness of the morality of his acts and thoughts. The education of the active powers comprehends the training of the organs of motion and of sensation.

*Education of the muscular system.* The simplest apparatus is alone necessary. A table, a balance, a ladder, and dumb-bells, (or more simply, the two latter,) are alone required. Exercises demanding sudden and strong exertion are not required, but rather the constant exertion of force during a fixed time. There are no idiots who are well balanced,—all have either a preponderance of nervous excitability, or of muscular force, or are in complete atony. The education of the nervous system must therefore go hand in hand with that of the muscular, and be regulated according to the idiosyncrasy of the patient. Idiots are deficient in muscular sense. Prehension precedes standing in all children. When the idiot cannot use his hands he should be put on and in front of the ladder, held by his belt, and his hands and feet directed to mount and descend. If his hands refuse to hold the rounds, let him fall into the attendant's arms, and be again replaced. If this is not sufficient, M. Séguin makes the child mount the ladder behind, whilst he mounts in front holding its hands; every step being made slowly and surely. In descending he disengages one foot which clasps the step, with his own foot with which he guides the child's foot to the next step. He disengages one hand which by an instinct of preservation, rapidly clasps the step below; by these means the inert muscles contract energetically, and support a weight and shocks which no voluntary exercise can accomplish. The same exercise may be repeated on the reversed ladder. As soon as the muscles of the hands have been taught by this instinct of preservation to contract, they must be applied to taking food, to useful purposes, such as handling stones, bricks, spades, wheelbarrows. Dumb-bells can then be used, and the balance (which is a bar of wood with wooden knobs at each end) in order to make the child stand still, and walk steadily. When the abdominal muscles are so weak as to prevent standing, the child may sit with his feet on a spring board, to be moved up and down. Some cannot even sit, and must be taught at first to sit still without any support. Before any regular action of the limbs can be obtained, the child must learn to stand still. To do this he may at first amuse his hands with dumb-bells, and fix his feet either in boxes, or (which he often prefers) in a round or square space drawn with chalk on the floor. By whatever means necessary, this power of standing still must be obtained, as it is an essential step in rendering the motions obedient to the mind. When the powers of motion are much impaired neither the capability of standing, or of ascending or descending can be acquired without great practice, varied according to the individual. All should be habituated to those uses of the fingers necessary to daily life, to dress, to button their clothes, to tie, to fold, to carry, to arrange, to wash, and comb, &c., and subsequently to be able to cut, to saw, to plane, to draw, to sow, plant, water, &c. The aim is not to make muscular prodigies, but to employ the muscles usefully, and for this purpose a modest system of gymnastics, and a master patient in little things, are wanted. It must be remembered that there are three kinds of defects of motion which may be met with,—one in which the muscular power is deficient; the second in which the power of the will is wanting; the third, defects of structure, as contraction or retraction. For the first, purely muscular exertions with or without the will; for the second, voluntary efforts duly regulated; and for the third, an orthopedic treatment adapted to the maimed limb.

*Imitation* is a powerful means of instruction. It is *personal* when its object is to modify the individual's own acts and habits, *impersonal* when it relates to his action on outward things.

Personal imitation. Those who have the faculty and in whom it is not directed contract grimaces, and tricks of expression. Such must be got rid of. The first step being to enforce muscular repose. This is done by the double operation of imitation and authority.

"A. H. was (says M. Séguin) indomitably petulant; clambering like a cat, escaping like a monkey; he could not be kept standing still for three seconds. I put him in a chair sitting opposite him, holding his feet and knees between mine; fixing his hands on his knees with one of my hands and with the other bringing incessantly before me his moveable face. We remained thus *for five weeks*, meal and bed-times excepted, after which he began to stand, and almost immoveably."

When immobility is obtained the exercises of imitation may be begun. The first lessons are to teach the idiot by gestures and word the parts of his own body, and their common uses. Then the difference between right and left. The next exercises are to make him move his limbs at the will of the teacher and by simple imitation, singly and then together; to close his fists, to open his fingers; to bend the index finger whilst the others are stretched out, &c. This personal imitation is more quickly taught in classes.

*Impersonal* imitation. There are three kinds of exercises.

1. The idiot is to repeat exactly the movements he sees his teacher execute who places objects in use in very different positions. The teacher takes a plate and places it on the table, indicating to, or telling the child to do the same with another. He then returns the plate, and the child imitates this: next places it vertically, &c., and puts it in numerous positions; and the same with a glass, a brush, a hat, and other objects in common use.

2. Place objects of no use in certain forms, such as wooden bricks as squares, triangles, &c., to be imitated by the child.

3. Make a vertical line on a board with chalk, which the child must imitate first by moving his arm in the direction and then marking. Next, a similar line from below upwards: then from right to left and from left to right. All idiots prefer their left hand.

### EDUCATION OF THE NERVOUS SYSTEM AND OF THE SENSES.

The senses are to be educated in the following order. Touch, sight, hearing, taste and smell, that is in the order in which they are awakened in the healthy child.

*Education of the touch.* The most injurious effects of the disordered direction of this sense is in that bad habit to which idiots are much exposed, often by discovering themselves the gratification derived from it, and not by imitating others. Some idiots have no consciousness of the sensation produced by touch, whilst others have neither the consciousness nor the sensation. In the former it is only necessary to awake the consciousness, whilst in the latter the sensation itself must be provoked before attention of the mind can be given. In the first case it is important to associate hearing and sight with touch; in the last this is useless, but the touch must be roused by energetic and varied shocks, by agents whose actions are opposite, such as heat and cold.

To educate the touch of idiots, it is often sufficient to give him things to handle and to distinguish without using his other senses. The exercises are: 1, With hot and iced liquids; 2, astringent, emollient, oily fluids; 3, resisting or elastic bodies; 4, bodies which are rough, smooth, woolly, cottony, or silky; 5, heavy and light bodies; 6, bodies of the same form but of different size; 7, of varied form. They should be taught to distinguish slight differences in heat.

*The education of the taste and smell.* The children often abandon their senses to all the vagaries of their curiosity: nothing is too strong, or acrid, or fetid, or astringent, for their uncultivated tastes and smells. Although these senses have no direct connexion with the intellect, there can be no doubt that the harmony and due development of all functions is important. As a rule, stimulants should be avoided; but if necessary cayenne pepper, mint lozenges, or very small doses of colocynth may be used to stimulate the languid sense, and when it has produced its effect it may be withdrawn. From these strong tastes the scale may be gradually descended to delicate ones. It is sometimes necessary to rouse the smell by long and frequent applications of ammonia or otto of roses, but in general the stronger smells are perceived.

*The education of the ear.* Very few are deaf, many however never listen, but in general the idiot loves and readily seizes rhythms, for the musical faculty is one of the gifts of idiots. M. Séguin has never seen idiots (except those absolutely paralysed) that did not express the liveliest pleasure at music, and he has known a great number who sang correctly though they spoke badly and with difficulty. One chanted all the vespers though he could not pronounce two words in succession. Another came out of a deep torpor during music. Another, generally torpid, experienced great pleasure in a concert; he smiled, his face became animated, his hair stood up, his fingers contracted themselves and were agitated, and his forehead and hands covered with sweat; after the last note he sank into his usual inanity. Another repeated difficult tunes after once hearing them, whose only articulate sound was papa, which he said with difficulty, imperfectly, and inappropriately.

Idiots are more alive to energetic, rapid and gay tunes, than to slow and grave ones; also to instrumental music than to vocal. The remedial influence of music has not yet been sufficiently studied by M. Séguin, to enable him to lay down rules for its application.

There are three kinds of exercises for the ear:

1. On sounds in general. The child must be taught to hear the sound of bodies falling, or of the contact of one sonorous body with another.

2. The gamut. He must be taught the differences in musical notes.

3. The voice as indicating the feelings. He should be made familiar with the expressions of joy, fear, and pain; in these exercises it may be necessary that the ear alone should be addressed, the action of the other senses being for the time suspended.

*Education of speech.* Some idiots are dumb from paralysis, perforated palate and deafness; but in general their muteness does not depend on want of voice, for the voice is strong; nor on the inability to articulate, as they can pronounce distinctly some words; but it consists in the few syllables or words they can pronounce not being spoken apropos, nor voluntarily, nor intentionally, and these accidental sounds having no sense. The idiot is said to be unable to speak, because he is unable to express his wants or sensations in words. This inability depends principally on two causes,—an incapacity to move at will and with ease his organs of speech, and that want of will, that repugnance to perform any spontaneous act which is the characteristic of idiotcy. The organs of voice should be carefully examined, as their structure is often anomalous,—such as defect of action of the muscles of the cheeks and lips, contraction or great width of jaws, atrophy, hypertrophy, or immobility of the tongue; great elevation or depression of the arch of the palate.

First exercise. When the child has been taught to imitate, his attention should be directed to his mouth. The index finger should be put across his lips, then horizontally, then between them, and then two or three fingers introduced into his mouth. Let him hold a ruler between his lips, if they do not contract increase its weight; if they are too far apart gradually diminish its size. Make him masticate hard food, next make him simply imitate the motions of the lips in speaking. If there is a want of proportion between the tongue and the palate, as the latter cannot be improved, the tongue must be rendered more agile by a system of tongue gymnastics. He should be taught to move his tongue out of his mouth, up and down and sideways, keeping it some time in each position. If this cannot be done by imitation or direction, the tongue should be directed with a wooden knife or ivory spoon.

Second exercise. The next step is the development of the voice. The principle M. Séguin follows, is to observe the natural course of this development and to pursue the same. Thus children begin with sounds like pa, ma, dada, bo—bo—mi—mi; not with a vowel but with *a labial consonant followed by a vowel;* and they love to repeat the syllable. Such sounds, (as they are much easier, than vowels alone, or than consonants preceded by a vowel,) should form the first lessons. Even this plan must be modified according to circumstances. Some cannot make a long and pure vocal sound, and must be taught by imitation to do this before they can articulate; and in some whose lips do not contract it may be necessary to begin with linguals before labials, as the difficulty of the latter will discourage them. Two cases are given which show the method more clearly than mere rules.

A. T. a hydrocephalic child, with a high, deeply-grooved palate, talked constantly, but could not articulate. The letters he could pronounce were N, as non, which he repeated many thousand times a day, M, as ma, and P simply. He could remember and sing tunes. As he could pronounce labials, he was first made to practise them, to distinguish PA from PO, and MA from MU. From P and M he was led to B, through BO and BU. V and F were next attained by placing his under lip beneath his upper incisor, teeth, and this led to the sibilants S, CH, Z. J. By elevating the tongue anteriorly LA was pronounced, and next the labials T and D, and thence to the gutturals. He completely acquired speech.

The second instance is of a child of 13, with a small circular head, whose faculties were seriously impaired but whose voice was good. He could imitate any syllable of two letters only, but could not unite two unless they were the same repeated. Thus he could say mama, but not maman. M. Séguin first made him repeat A, following it with a syllable of two letters, as A-TA; next, A, O, which after some time he accomplished, and seizing a moment of excitement his teacher added T, so as to make it A, TO, a double articulation previously impossible, then A-DO, A-LO, &c., then the labials; next two syllables, keeping the first syllable and second vowel constant, and only changing the second vowel, as TA-LA, TA-BA, TA-RA. Next changing the second syllable wholly, as TA-BO, TA-VO; then changing the first only—then changing the vowels only. For syllables of three letters, which were next attempted, the two first were kept as constants, the last changed. The task was long and painful, but the child spoke distinctly at last.

As this part of education is the slowest and most difficult, it should not be begun until some power of imitation has been attained; and all the exercises must be practised a long time; analogous syllables must be first united, then different ones, then whole words, until those of the most difficult pronunciation are articulated. Stammering often hinders progress. Its remedy must depend on the circumstances of the case, and on this adaptation to the individual case M. Séguin attributes the benefits which has followed his means.

*The education of the sight* requires great and methodical attention, from the difficulty of acting on the eye, which is an active organ so situated as not to admit of being mechanically directed. The first point is to fix the vacant wandering eye. Three exercises may be practised: 1, Shut the child in a dark room in which is a luminous point that can be moved so that the eye may follow it; 2, Throw the balance from one to another, so as to attract the eye to follow it; 3, Place the child before you, and follow his wandering eye with a firm and obstinate look which will provoke attention: the intelligent and animated eye follows the inactive eye, arrests it, fixes it, and then directs it. But this most important exercise is often very difficult to execute. The child avoids your look, and perhaps closes its eyes. M. Séguin attempted in vain for four months to attract the look of one child. The first time the child's eye met his own, it uttered a cry; the next day, instead of mechanically feeling him, it looked at him as something new; and day after day renewed this scrutiny with intelligence until its curiosity was satisfied and it saw without astonishment. By speaking to the child at the moment its sight was roused, it looked at him afterwards when spoken to, and thus its hearing improved. M. Séguin has found that the progress of the idiot is not so appreciable during the education of one faculty, as during the transition from one exercise to another.

Direction of the eye being obtained, even although imperfectly, it must be taught to distinguish colours and form. Square octagonal cards of different colours should be used, and square cards, of two colours (say orange and blue), being placed on the table, two octagonal cards of the same colours should be given to the child, who should be directed and commanded to place each on the square card of the same colour, and the colours should then be named. To give a notion of length and size, many pieces of wood of different lengths and sizes should be procured, and when placed together, the child should be made to point out the longest and shortest, and the biggest and least, beginning with those whose differences were very apparent. For forms, it is at first necessary to have holes corresponding in size to the circle, square, or triangular model, into which he may fit it; afterwards he must be taught to place one moveable shape on others merely painted or drawn on paper.

*Arrangement* may be taught by pieces of board or wooden bricks. Give one to the pupil, and taking another yourself place it in various positions, making him place his in the same; proceeding to two, three, four. Build bridges, houses, &c., with wooden bricks. To understand *plans* is an important step. The simplest forms, as the circle and square, should be begun with, and the child taught to carry its finger round their edges, and to put it, following the teacher's, on each of the angles. Next, the teacher, placing his finger on one square of a chessboard, must direct the pupil to place his on the corresponding square. The same may be done with figures of houses, animals, soldiers. Thus the child is taught to direct his hand to certain given points.

*Images* are useful, but they should be at first representations of simple and common objects; lines, figures, houses, carriages, animals, and finally groups. As soon as an idiot takes pleasure in these representations, they should be multiplied; his taste cultivated by seeing good pictures and statues in museums, and he should be led to give an account of what he has seen afterwards. Those of M. Séguin's pupils who have made most progress are those whose intellects have been most trained by this examination of works of art.

*Drawing.* In order to draw, he must previously have been taught to distinguish the topography of a place, to know what is above, what is below, the left from the right, and to be able to place his hand or finger on any given point, and to direct it; for he is unable to do any of these things. To draw a line in a given direction is the first step in writing as well as drawing. Children acquire these notions generally by intuition; but every step must be carefully taught to an idiot. Having mastered these principles, the first step in drawing is to draw a vertical line between two points; but as the hands of some descend to the right, or left, or wander about the paper, it is often necessary to draw as guides two parallel lines, one to the left and the other to the right of the points, or besides this to fix two vertical rules so as to prevent the hand from deviating. Gradually these helps are to be withdrawn until the child can draw the line even without the points. The same method must be followed for horizontal lines. Curves are to be first drawn on these lines. Having made a vertical line, draw a curve, beginning at its summit and ending at its base; the child sitting at your left, and the curve drawn from left to right, by which means the hand does not hide the course of the line. A similar curve is to be drawn on a horizontal line, joining the lower end of the vertical. Four such curves form a circle. In uniting lines to form figures, M. Séguin has found that the idiot experienced great difficulty in drawing a square, but made a triangle more easily. He therefore begins with a right-angled triangle; joining his vertical and horizontal by an oblique line. Four such triangles make a square, the inner lines may be effaced, and afterwards the square drawn simply. Curves on each side make a circle. From these simple steps the idiot learns to make the most complicated figures, but the process is a laborious one for both parties. M. Séguin incidentally mentions a fortnight being required to teach one child to make a straight line. When the motion of the hand is irregular and jerking, it may be necessary to make him draw thousands of circles from right to left and left to right. Sometimes by drawing a white circle on a black board he follows the mark; or one circle within another, and between which he draws his own circle; in others, two projecting circles are necessary to guide his hand.

From drawing these geometrical figures, the idiot passes to forming letters. Thus D is only a half-circle resting on a vertical line, A two oblique lines united at their summits, and divided by a horizontal line. Having learned to draw, he can write. The letters must then be traced according to contrasts and analogies—O next to I, B to P, T to L, &c.

*Reading.* The exercises in plan, colour, arrangement, size, and form, are necessary steps to understanding letters, and the notions of which they are the signs. The next proceeding is to learn the names of the letters. A frame is constructed, in which are placed each letter of the alphabet painted on a card, to each of which a metal letter exactly corresponds. Two or three metal letters are placed before the child, and he is directed to place one at a time on the corresponding letter in the frame, the teacher naming them. Subsequently, a letter being shown to him, he is told to name it: if at first he were told to name it as well as place it, he would be puzzled. He thus connects a name with a figure, and a figure with a name. The order in which the letters should be taught to be pronounced is,—labials, labio-dentals, dentals, linguals, gutturals. Letters being understood, syllables are to be mastered, or the relation between the sound and many signs; and also the relation of many signs with many successive articulations. Here the previous exercises assist. The idiot who has placed two bricks to form one figure, is led to comprehend how two letters can form one sound.

*Words.* The next step is from mechanical to intelligent reading. To connect words with objects and ideas. For this purpose the card letters forming the word (bread, knife) should be placed on the object; then the object should be given to the child, who should find its name among several names placed before it. The child at first should never read a word without understanding it, and therefore it should be the name of an object of sense: thus all the persons and things about him should be named, and alternately written and pronounced.

*Notions and ideas.* M. Séguin has carefully distinguished between these two words as the basis of his plan of education. He explains the different sense which he intends to convey by an example. A child when he can distinguish a key from a table or a hammer, has the *notion* of a key; but he has the *idea* of a key, only when he knows the relation between the key and the lock. The *idea* is the result of the mind's operation on two notions, key and lock. It embraces their relations, the reason of these, and their consequences. By the *notion* of the key the child distinguishes the key from other keys and other things; by the *idea* he understands its use. By *notion* he means to convey merely the knowledge gained by the senses; by *idea* the result of reflection on these impressions. The first appreciates the physical condition of bodies through the senses only; in the second their relations through the intellect. *Notion* is passive, *idea* active. *Notion* is acquired by the senses. *Idea* by the reason, through deduction and induction. It follows that education must commence by notions which embrace all phenomena which are objects of the senses. Once acquired there will be materials for *thought,* which, however, cannot be taught.—You may give the child notions, but his ideas must spring "proprio motu."

*Practical grammar.* The mode in which substantives are to be learned has been stated: the next step is the expression of their properties by *adjectives.* The child should be placed before a table, and taught that it is brown, round, a square, on two or four legs, so as to establish the differences between it and other tables. At first the object itself should always be shown to the child, and the words expressing its properties written. Subsequently names with their qualities (as a white horse, a sharp knife,) should be written, and the child point out the object among many, and so on. Action is expressed by the *verb,* which should be shown by sensible images. At first read or write the verb expressing some of the instinctive actions, as eating, and interrupt the act of eating to impress it: next, having written "to strike the table," the master should point out the verb, and imitate the motion, and vice-versâ. As to the tenses, their order must depend on the language, and the rules for the French will not wholly apply to English. He begins with the infinitive, then the imperative, the conditional, the present and the future. The preposition comes next. Having written "put the bottle *upon* the table," the child does it, then "*under* the table," he obeys, &c.

*Memory.* In the education of idiots no words should be taught which they do not understand. The first exercises should relate to sensible things: the idiot should be requested to bring some object from a distance: then two, then three, then more which he should choose from many others: next he should receive the same orders for things in another room, and next for a time at some little distance, as for five minutes, half an hour, a day. Afterwards he should be ordered to perform personal acts, to wash his hands, at a certain hour before his meals; to remember the time of rising or going to bed; to say at noon what he has done in the morning; and in the evening what he has done during the day. From notions he may pass to ideas; but he should never be taught to repeat what he does not understand.

In those who have no memory the following plan may be tried. After the idiot has seen his food, remove it and ask him what it was; after some trials this will succeed. Afterwards let him name the food he has eaten. From thence pass to what he has seen, heard, &c. The real use of the memory should always be remembered. It is to furnish the mind with materials from the past to enlighten the future. Its final use is for foresight, and it therefore leads to a taste for useful labour, and to that positive knowledge which is of practical utility.

*Provision and foresight.* The most trifling occurrences of the day afford lessons in these important acts of life. The child wakes and he has no shoes, for he has forgotten over-night to order them, which was his fault: he has not got what he likes for breakfast, as he had not ordered it before; this is his fault: he has no money in his walk to buy refreshment, as he forgot to bring it. It is his fault, always his fault. This part of his education is most important; for without foresight he is the victim of slight circumstances and of all his wants.

*Arithmetic.* Numbers must be taught by objects; any will answer. M. Séguin makes use also of balls like billiard balls, numbered, with holes for them having similar numbers. To learn the numbers is an affair of practice and memory; some little help may be given by rules, a few of which are given. *Calculation* requires thought. M. Séguin takes *two* as the basis. He makes the child take off his shoes. How many shoes have you? Two. How many pairs is that? One; and so on with 2, 3, 4, and 5 and 10 pairs, and the odd numbers varying the objects at each lesson, and applying the numbers to quantities, weights, dimensions, size, distances, capacities, and at last to value; teaching by things and not by abstractions, and thus educating by means of the senses. In some idiots there is a natural faculty for calculation, but as this is useless alone, it should not be cultivated at the expense of the other and more useful powers. The principal application of calculation necessary for idiots, is to understand the use of money. They must be first taught the difference between the different coins, their form, colour, weight, substance and name; next their use. This can only be taught by practice. They must see others pay for goods at shops, and be taught to purchase themselves, by first buying some sweetmeats. M. Séguin gives the details of his management in shops with his pupils, for this, which is a simple amusement for other children, is a study for an idiot. He has among his pupils, idiots whom he has taught to count, keep and spend their money, to go to market, to the butcher, &c., and for whom their mother's care becomes every day less necessary. For such he has attained his full object in teaching them calculation.

*Natural history.* The idiot should be taught the names, differences in form, colour, and the use of these animals, and also to take care of them, to feed them regularly, and to clean them. This gives him foresight, address, and neatness. Horses, sheep, cows, and such animals are all lessons for him; and he should also be taken to see wild animals. Vegetables are means of instruction: he should learn to distinguish one flower from another, by their colours, form, and smell. He should be taught to sow, plant, water, and gather. To teach him *cosmography* by maps would be impossible at first, but his teacher, with a rule always in his pocket, should constantly instruct him in distances, and in sizes, the longer and shorter, the less and the greater. He next should compare those which are separated: as the length of a room with its breadth, or the size of one piece of furniture with another; next the relative length of the garden and the court, and their relative position to the house, and the house to the street. One village is the end of a short walk, another of a long walk; in what direction are they? From this actual topography of the town and neighbourhood, the teacher passes to maps of the county, making the town the centre, and teaching the pupil to trace his journeys to certain places and back again. Their astronomy consists in learning that the sun rises before such a window and sets before another, &c. The great art in his education consisting in limiting him to positive and not speculative information; to facts such as can be presented to his senses, and not abstractions which to him are mere senseless words.

*Education applied.* The object of this training is not only to develop the faculties of the idiot, but to apply them to the common actions of life.

1. *Decency.* 2. *Bodily habits.* All tricks and unseemly bodily movements should be carefully eradicated. How necessary such training is in ordinary children! 3. *Attitudes.* The idiot lies in bed like a ball, or throws off any clothing, turns his head from side to side for hours; all such habits should be overcome, sometimes with mildness, sometimes with energy. He rarely sits well, and this becomes a cause of deformity. He should be made always to sit on a stool without a back, or arms, and the height of his knees. His bed should be hard; he should not lie long in it, nor sit much. Standing still is the cure for many of his ungraceful motions when erect. 4. *Walking.* He walks with the gait of a drunkard; and requires careful drilling. This unsteadiness prevents him from going up and down stairs with any ease. He is obliged to bring both feet on the same step before he moves one, and he always moves the same foot first, and with fear and trembling. Much practice on a light staircase with low steps is necessary. The gymnastic belt furnished with two rings, one in front and the other behind assist the teacher. 5. *Dressing.* The idiot should first know every part of himself, and be taught next to name every article of his dress, and to show to what part of himself it belongs; next the order in which they are to be put on, and finally the manner of putting them on. These explanations require long efforts, and the complexity of the common operations of lacing stays, putting on braces, is seen by the difficulty. The teacher requires great skill as well as patience. 6. *Eating.* He should be taught to eat alone, and neatly, masticating well. When he has given signs of appetite, the attendant should eat before him slowly and deliberately, performing each step in the process, chewing vigorously, and placing his mouth so near as to show the food between the teeth. His attention being thus attracted the attendant should eat, sitting opposite to him at a narrow table, putting a spoon, or fork, or knife in his hand; if he is slow, be slow with him, making him take every spoonful, and mouthful, and draught in imitation. When his appetite is satisfied, play with him and induce him to speak and act freely, when he will often declare his tastes and habits. Hard and stale bread which he must masticate to swallow, is an exercise tending to strengthen his organs of speech. M. Séguin doubts whether he has sufficiently insisted on the importance of proper habits, as bad habits in idiots are so numerous from their idleness, and their infirmities. Patience and resources are necessary in the teacher, who should always seek the simplest and most natural remedies. Almost all manual exercises are useful. 8. *Useful tastes.* Constancy in quiet, agreeable, and useful tastes renders the idiot bearable to others. Such habits are the fruit of education alone. The kind of tastes must depend on the station of the child. The manners and gestures should be taught to approach as nearly as possible to those among whom the idiot lives, so as not to disgust; and to render their tastes useful, they should be taught to work. 9. *Useful work.* This is most essential to the poor, as the neglect and brutality with which they are treated is often from their expense and uselessness. But besides this, work is essential to health and morality. It is the fulfilment of duty. "Wherever I have gone (says M. Séguin) my object has been to organize work. In public institutions, field and labouring work, and joinery; at home, needle-work and household work for girls; and trades for boys. Simple and useful work on which their health and morality depend."

### FIFTH PART.—MORAL TREATMENT.

M. Séguin confines his observations to morality as embracing the relations of a human being to others and to himself, and not including those with his Maker, the higher department of the clergy. In many cases the teacher may assist, and M. Séguin shows his own religious feelings by giving two short prayers which he makes the idiots of the Bicêtre repeat before their meals and their exercises, praying for God's blessing on them. Morality then, as including the regulation of the actions, intellect, and passions, is essential to any education. The child's will must be subjugated to the authority of another's will as the first step.

*Authority and obedience.* Idiots are less deficient in the power of commanding others than in obeying them. Obedience irritates them, authority fatigues them. Some employ the best devised artifices to bend another's will to theirs; others use ten times more skill in escaping an order, than it would have required intelligence to have obeyed it. Another would obey a man passively, though it would enslave a woman, highly intelligent but without authority.

"Miss ——, (says M. S.), at eight years old was intrusted to me; she had subjected all her family to her orders. Though an idiot and mute she saw at once that her relations were changed, and she obeyed. A year afterwards her mother visited her, and she began tormenting her, and disobeying me in her presence; I made the mother retire so as to see unobserved, the implicit obedience of the child to myself. Here the cure was not complete. Subsequently she became obedient to all her family."

An idiot, as well as children generally, perceives sooner than an adult the degree of authority to which they must yield, or which they can overcome; they do not struggle in vain, but know even by a gesture the will that can overcome them. In obedience to a well-regulated command their physical health depends; the child badly governed, sometimes with feebleness, at other times with force, or with indulgence, does not fear such versatile authority. He infringes, or eludes orders: becomes cunning, irritable and violent, and these mental irregularities bring indigestion, interrupted sleep, and nervous disorders.

That the idiot should obey, his teacher must know how to command, and be able to apply all means of authority from the most rigorous to the most insinuating. Obedience is not a simple affair; a child who for fifteen years has only learned to obey his masters who are with him, has often only learned to be disobedient the rest of his life. There are degrees to which he must be gradually submitted. 1. Obedience to his master when present. 2. Obedience to orders which may be immediately enforced by the presence of his master. 3. Obedience to an order, in the absence of his teacher which leads to obedience to a moral principle assented to by the reason. Or, in genuine English, obedience to a sense of duty, and we thank God heartily that we have in England in daily and in hourly use a term which does not occur in this whole volume, but which foreigners are forced to admit is the strong characteristic of the British character—*a sense of duty.*

At first *simple* obedience must be enforced. The idiot must be made to look, to touch, to act, to perceive, to compare at the will of his teacher. He must be at first passive: his progress, and the use of his own free will is the fruit at last. For the end of education is not passive obedience but liberty, and to be free there must be a will. *Liberty* and *will* are words only to be spoken by those who comprehend *obedience* and *authority.* No one can command but he who has learned to obey. The *will* regulated by the intellect and the moral sense is the secret of success in men of character. *This* will is the result of education. The idiot is destitute of it. *His* will is governed by his instincts, and by his inertness. Indulge his appetites and let him alone and he is content. But moral education consists in conquering these appetites, by breaking up his habits, and in subduing his indolence by varied and incessant activity. The instinctive and negative direction of the will having been subdued, the will must be morally trained by imitation, by authority, and by compression; it grows morally by the *resistance* given by a good education to the first instincts.

*The master.* Every one cannot teach idiots; the teacher must have a will, he must have the faculty of command; this is by no means in proportion to the intellectual power; the intellect and will are distinct faculties, and this is an important principle in M. Séguin's method. Often the labours of the study seem to deprive a man of the personal power of influencing others. A teacher should have no physical defects, but above all moral qualities he should be *calm*—whether exercising firmness, energetic decision, gentleness or insinuation, under all his aspects and in all moods, *calm.* Calm in command, calm at table, calm at play, of an unchangeable serenity of mind; if he has never had an occasion to exercise that self-control which produces this calm, no better opportunity of self-conquest than the education of an idiot can be afforded, for no other occupation demands more patience, more observation, more concentrated action, more calmness; but a man capable of dedicating his youth to the education of an idiot is found with difficulty, and one who gives his life to the work must daily call to mind the sacred words, "the good shepherd gives his life for his sheep."

But how is the idiot to be ruled? If he is capable of foresight he has been apprized of the new hands into which he is to be placed, and looks forward with some anxiety, so that the first interview is well calculated to impress him; but for this purpose the master should act as a master, not as an ordinary visitor; his address frank, his language and gesture plain, and his manner so decided that at first he is remarked, listened to, regarded, and recognised. If the first impression succeeds and has not been weakened by the comments of relatives and servants, the master has gained and will keep the magisterial position necessary to his success; but he must not think that time will increase his authority; he who looks to time has had little experience either of men or of idiots; if he is really the master he may in time relax his authority as the pupil's own will grows and is more regulated; but if he has not gained the ascendancy the child does not lose his time, but hour by hour, day by day, will steal from his master some concession, surprise him in some weakness, will profit by the lassitude of one day, and the indisposition of another, to command him in his manner; that is negatively, in doing or not doing, whatever suits his disordered instincts. The master therefore must at once take up a decided position, raising his authority above that of the family, which has been weakened by daily concessions to a delicate, suffering, or ungovernable child; the weakness of parents must be pitied, perhaps, rather than blamed, but the master must not for a moment hesitate as to how he is to act. If he cannot be master, he can do nothing.

*The pupil.* This necessity of commanding the idiot depends on his want of a moral will. Accustomed to do nothing, he will do nothing; used to manage his parents, he will attempt to manage his master, by force or address, sometimes by smiling and looking at him with that scrutinizing eye which sees into dispositions; sometimes by anger, or by crying, biting, rolling on the ground, or escaping from any constraint. From the energetic—no! no! incessantly repeated, to the most subtle pretences of fatigue or suffering, the master has often to be exposed to all the various batteries of negation; he must expose to the family the nature of this resistance, for its energy proves the necessity of control, and that a will is present but not exerted in the right direction. The struggle between the two wills, the master's and the pupil's, may be long or short, ending by the conquest of one; the pupil will conquer if the master has not the faculty of command, and especially calmness: or if the parents take the child's part against the master.

*Immediate command,* or that of the master when present. Resistance varies, and rules must be modified: those given, suppose the maximum. To make an idiot will, it is necessary first to will for him, to will that he should will; the command given in the most imperative manner, with the words supported by gesture and look. If the master's voice is dull and insonorous, if his articulation is vicious, his words dissonant, nasal, long and monotonous, he labours under great disadvantages; he should unite the two qualities of a flexible voice and a perfect articulation; his commands should be in short, sonorous, and decisive sentences; in the explanatory part the expressions may be slow, detached, every syllable expressing its meaning aided by gesture and look. All the notes of intonation and articulation should be contributed, from the short, dry command, to the most sympathetic and fascinating intonations.

*Gesture* assists. It is a constant excitement to imitation and activity. To attain a facility the master should practise privately with another to express by signs only what he wishes; the master should also do himself what he wishes the idiot to do. The general rules are, that gestures to enforce stillness and attention are made by moving the hand from above downwards, and from without inwards; those from below upwards, and from within outwards, are used to excite activity. Again, the movements of the hand and arm should be in a straight and rapid line in imperious commands, and in a curved line produced by a slow and undulating movement in explanatory commands.

*Expression.* The eye in fixing attention becomes a powerful instrument; the idiot should feel that it is always on him, and that it expresses better than words whether his master is satisfied or not; it should command him at first, and afterwards guide him, animate his activities and sustain his exertions; for this purpose the master should not only have a good eye, but should have studiously practised its use. As his gestures and words should be more decisive than ordinary, so also should his expressions; he is not to stare and alarm by making faces, but simply to express by his eye and features what he himself feels at the sight of a child when obedient or disobedient, attentive or inattentive, patient or angry, and to make their expressions so marked that the child cannot mistake his meaning. For this purpose,

1. The physiognomy should be naturally moveable, expressive, and inclined to reflect the feelings. 2. It should always be in harmony with the mind. 3. The expression of satisfaction should neither be long, lively, nor noisy, so as to interrupt employment, especially if the idiot is vain. 4. The expression should be confined to the thing in hand, and never be interrupted by what is foreign to the immediate purpose.

*Coercion.* No other means but words, gestures and looks, should be employed. All children are born with a spirit of resistance, children are all of the opposition; no, precedes yes by many months. This instinct of negation is most marked in idiots, and when joined to their extreme laziness, and confirmed into a habit, by finding it overcomes the reasonable wishes of the family, it must not be expected that the master can at once overcome it. No! he will be opposed in every way; but let him will with patience and perseverance, and the child will obey him. If, however, he has a pupil who has this habit of negation, and has ruled his parents, the master should never begin with an order which can be refused him. He must begin by negative orders, chiefly as—not to go there, not to touch that, or not to eat this, and by positive orders of which he can compel the execution, and afterwards proceed to those which demand a concurrence of the child's will; the transition requires an adaptation to circumstances which makes it a scientific art in the hands of a man gifted with that serenity of mind and charity of heart necessary to success.

In cases of extreme resistance the master must put himself in immediate proximity with the pupil. For instance, in commanding him to raise his dumb-bells he only raises that arm next to his master; he must therefore place himself before him beginning and continuing the action for him, and thus acting immediately by word, look and act on every part under command; this is hard and painful for both, especially for the master, but it is not necessary that both should be incessantly together: a milder authority, a half-will in the intermediate times may carry out the plans, a woman attendant, who knows how to resist the perverse will of the idiot without completely understanding how to enforce her own, is sufficient. The imperative command is necessary for each new step, but the child would repeat better the new lesson under a less rigid disciplinarian; the master passes from this imperative mood to a milder form or *mediate* command.

From subordinating the idiot's will to his own, the master next induces or excites him to act for himself. The favorable moment of transition must be watched and seized, it cannot be hastened; it is infallibly indicated by two signs usually simultaneous: the absence of all resistance to authority, and some spontaneous wish for an active and intelligent occupation. An instance is given of an idiot who would do nothing but keep in incessant motion; the usual steps of compelling him to be still and to use certain exercises were gone through; one day he seized a crossbow which had been used to direct his eye, and he shot the arrow through the eye of one of his ancestors in his sculptured frame. His governess was in despair. M. Séguin was enchanted; he procured painted lithographs of heads, stuck them along the garden wall, placed the crossbow at hand; the idiot seized it, discharged it at one of the figures, was encouraged, did it again, and subsequently the promise to repeat the play kept up his attention and patience in his exercises.

After the first spontaneous act of an intelligent kind the immediate imperative authority should be gradually discontinued, and the idiot should be induced and excited to obey, through the means of his tastes, preferences, ideas and feelings, which the master understands; gradually spiritualizing him; commanding him more by words than gestures—more by the sense of words than by the sound of the voice—more and more by hopes, reasons, and feelings. This is a very gradual process; it begins by commanding the idiot to do a thing which he has done well, by an imperative order in a less imperative tone; diminishing little by little the rigour of the voice, the precision and decision of articulation, gesture and expression, until it is relaxed to the ordinary usages of society; but still the pupil must always feel that the old master can reappear—he must fear the claws beneath the velvet paw.

Stimulants of the tastes and desires are numerous. The idiot loves his parents and desires to see them; he likes the country and desires to walk; he likes flowers, cakes, pictures: all these may be promised as rewards. His antipathies may be used also as preventives of disobedience and idleness; but to be efficacious these promises must be facts—something positive and actual. He should see, or touch the object desired or feared; gradually the conditions should be adjourned, and thus his desires spiritualized by putting their satisfaction at a distance.

Games hold a high place in this education. M. Séguin mixes with his pupils as their playfellow, though he imperceptibly directs their games. A game is the most spontaneous act of infancy: it is more, it is the free and voluntary accomplishment of a bodily and mental function; an idiot who can play almost deserves another name. The choice of games lies with children, their variation and graduation with the master, who should take care that the game does not become a mere routine, but that there should be always something to learn; at first the games which please his tastes, afterwards those most useful. Thus when there is inability to direct the eye, the bow and arrow; for difficulty in motions of the hand or involuntary contractions, the battledore; for unsteady gait, the wheelbarrow, &c.

*Negative command,* or that which is not expressed in words but depends on such arrangements as shall lead the idiot to think and act for himself; the master has only to watch quietly and patiently after making his plans; the master has at first forced him to act, next has assisted him, now he places him in such circumstances as to force him to exercise his attention, comparison, judgment, reflection and moral will. He has arrived at that point in his education when he can execute all voluntary movements; he can read and write, whatever his master wills energetically he can do; but still he is an idiot, *ιδιος*, 'solitarius,' alone! He cannot act on persons or things by his own free will; he cannot spontaneously will; before acting on persons he must begin on things, and as the prehension of food is one of the first voluntary acts, the master may begin to exercise the will with this. The cloth is laid; you sit down, so does he, but the dish is wanting; you take no notice; he calls the servant, "Where are the chops?" You wait, he goes to find the dish. At first it is placed on the sideboard, where he can also smell it; another day in the passage which he has passed through; finally, in the kitchen; the same with the bread, wine, &c. Next, the dish is there, but no plate, no fork, no spoon, and he is compelled in the same quiet way to exert his own thought and seek them. Thus (if his health permits) a meal may last for hours, but the time is not mis-spent if he learns to think. At a later period he must have nothing for dinner which he has not ordered in the morning, or purchased himself beforehand. Dress is another stimulus to thought, especially as regards cleanliness; he must not walk unless he has ordered the washerwoman to send home his clean linen.

To establish his relations with persons he must have some want or wish that they can satisfy. When therefore he goes to find an object, a third person must detain it and exchange it for some other object which the child can comprehend, and which is not injurious. The simplest relations with others depend on the sentiments of property and of resistance; the inert, inoffensive idiot, incapable of defending himself, should be encouraged by innocent struggles which develop his strength and excite his confidence and courage. With regard to property, they should be taught that some things are common to all, as their water; some personal, as their clothes; some are both, as playthings; but as all have their equivalent in money they should be taught its use, among themselves; this will reveal indifference, prodigality, theft, and avarice, which should be corrected in time. The essential point is, that the idiot should cease to be isolated, that by his wants, tastes, habits, attractions, repulsions, he should establish voluntarily the most numerous relations possible with others.

The pupil has acted, perceived, and thought for some time, but he willed nothing spontaneously: he was motionless from a languor of the soul. Now his spontaneous will has been restored to him, and he has gained the energy that distinguishes man.

In conclusion, M. Séguin says that he does not regard his book as complete, but only as a commencement: two things are wanting to make the moral treatment not of idiots only, but of the insane as scientific and humane as it should be. The first is, the concurrence of men of *heart;* the second is, the solution of the great problem of education: this he thinks is at hand; for that society will no longer be content with mere education of the memory, or of the intellect, but will demand an education of the whole being, of all the functions of his body and the faculties of his mind, of his aptitudes, and of his artistical and moral sense; for an education of the functions such as Rabelais, Montaigne, and Rousseau indicated, does not yet exist.

On making the preliminary survey of the volume which we now close (consisting of 730 pages), there was no doubt as to the method of treating it. To be of any service to our readers it must be analysed. We have not criticised, from feeling that to criticise a plan of education which has produced such results, would be impertinence. We have therefore preferred the humbler task of clearly making out the author's meaning and giving it an English dress, and on this we have bestowed no little pains, and no little compression. Our analysis is the spirit of the book and not a literal translation.

The principle which guided M. Séguin, appears to us most sound and philosophical. His master was the celebrated Itard, by whom the idea of educating an idiot methodically was first put in practice, and to whom M. Séguin gives the credit for the first germ of his discovery. The well-known savage of Aveyron was the man whom Itard endeavoured to educate. It will be remembered that this was he whom the "philosophers" of Paris, during the revolution, regarded as a true type of an unsophisticated natural man. Disbelieving the Mosaic account they imagined man was created a savage, and rose by civilization to such a philosophical height as Paris then furnished. This savage from the woods was just what they wanted to prove their theory. But Itard failed in educating him. He was an idiot. And why did he fail? From the influence (says M. Séguin) of the false metaphysical system then in vogue. He and the then fashionable school imagined that the mind consisted alone of impressions on the senses. They did not recognize the existence of the mind itself. Itard therefore thought that education consisted only in the education of the senses, and he failed in making the savage a man. Séguin is a disciple of a higher school of thought. He has lived in a better period, when French materialism has been modified by German and British spiritualism. The former school asserted that there was nothing in the intellect which was not previously in the senses; the deeper thinker admits this as a half-truth only, but adds, that besides this, there is the intellect itself. The difference between the two principles is practically immense. Educate the senses and you educate the mind, say the one. M. Séguin and his school say, you do no such thing; you must, it is true, educate the senses, but if you stop there you have done nothing, you must rouse the mind itself to act; as long as it is the mere passive organ for the reception and remembrance of impressions, its *human* power has not been called out: the individual is not educated. But how does it act? By means of the will under the influence of the reason and moral sense. This moral will is the distinctive attribute of man: it is this which the idiot has not the power of exerting, and this deficiency makes him an idiot. To educate not merely his senses, but to rouse his free will to activity is the end of all education. Guided by this principle, M. Séguin has framed a system of education, and has successfully put it into practice. He is modest in stating the results of his plans, but we quote the following from Dr. Conolly's admirable letters on the Lunatic Asylums of Paris, published two years since in this Journal, describing his visit to the Bicêtre.

"No fewer than forty of these patients were assembled in a moderate sized schoolroom, receiving various lessons, and performing various evolutions under the direction of a very able schoolmaster, M. Séguin endowed with that enthusiasm respecting his occupation before which difficulties vanish. In all these cases the crowning glory of the attempt is, that whilst the senses, the muscular powers, and the intellect have received some cultivation, the habits have been improved, the propensities regulated, and some play has been given to his affections; so that a wild, ungovernable animal, calculated to excite fear, aversion, or disgust, has been transformed into the likeness and manners of a man. It is difficult to avoid falling into the language of enthusiasm on beholding such an apparent miracle." (British and Foreign Medical Review. Vol. xix, p. 295.)

We take leave of M. Séguin with respect and admiration. His book increases in real interest as it proceeds, and he gets into that part of his subject where he is indeed the master, and he transfers to the reader some of that warmth which he himself feels. We have a great respect for enthusiasm and enthusiasts. Nothing great, nothing heroic, nothing largely unselfish, nothing which proves that man is indeed of a divine nature, has ever been done without it. Our own Harvey and Jenner were, equally with Howard and Clarkson, driven by that inward impulse towards their mighty objects, which their duller contemporaries regarded as akin to madness. Many enthusiasts have, it is too true, been led away by a false and delusive light, but where, as in this instance, the path is one requiring unwearied, laborious, and self-sacrificing exertion, and the result not brilliant but silently humane, there can be no doubt that the guiding beacon is not the phosphorescent exhalation of a swamp, but a light from Heaven. We have aided in handing on the torch, and may it be the means of kindling a similar flame in the heart of even *one* earnest man, and of leading him to the amelioration of the sad condition of the numerous idiots in this country?

---

